# Creating animated medical images (Part 2)

**DOI:** 10.2349/biij.2.3.e46

**Published:** 2006-07-01

**Authors:** NA Kadri, MG Raha

**Affiliations:** Department of Biomedical Engineering, Faculty of Engineering, University of Malaya, Kuala Lumpur, Malaysia

## INTRODUCTION

In part one of the tutorial on creating animated medical images, the focus was on proprietary software, such as, MS PowerPoint (Microsoft Corp., Redmond, WA, USA) and Adobe ImageReady (Adobe Systems Inc., San Jose, CA, USA) [1] available in many personal computers (PC).

Here, in part two of this two-part tutorial, the procedure for creating animated GIFs using a selection of shareware and freeware will be described. The user may utilise the sequential CT images from the first part of the tutorial, but an additional set of images is also available for download at: http://www.biij.org/2006/3/e46/images.zip (1.01 MB).

## SOFTWARE

There is a lot of software, both free as well as commercial, to create animated GIFs. The majority of software available is shareware, where a user may download a trial version for free prior to purchasing [2]. According to Download.com, the two most popular software for creating GIF animation are Ulead® GIF Animator^TM^ (ulead Systems Inc., Torrance, CA, USA) and CoffeeCup GIF Animator (CoffeeCup Software Inc., Corpus Christi, TX, USA) [3]. Both have received high rating from reviewers and users.

In addition to these software, this tutorial will also describe a freeware, Microsoft® GIF Animator (Microsoft Corp., Redmond, WA, USA). All software used in this tutorial is available for download at: http://www.biij.org/2006/3/e46/software.zip (13.5 MB).

## ULEAD® GIF ANIMATOR^tm^

This software is a part of a multimedia suite of products developed by Ulead Systems Inc. (Torrance, CA, USA). For this tutorial, users may either download the software from the above link or download the current version from: http://www.ulead.com/. Users may try the software free of cost for 15 days. The current version, Ulead® GIF Animator^TM^ 5, is priced at USD 49.95.

Step 1 – Open Ulead® GIF Animator^tm^, and click on **File > Animation Wizard…**

Step 2 – Set the canvas size or dimensions of the image to be used. For this tutorial, set both the width and height at 512 pixels. Click **Next >** when finished.

**Figure 1:**
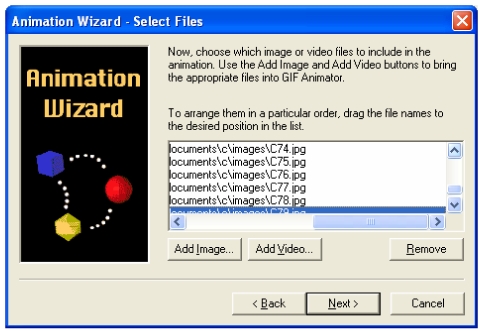
Selecting images to be used in animation.

Step 3 – Click **Add Image…** to add the images for use in the animation. Select all images (by clicking **Ctrl+A**) from the selected folder, and click **Open**. The list of images will now appear (Figure 1), and the user has the option of re-arranging the order of the images. Click **Next >** when finished.

**Figure 2:**
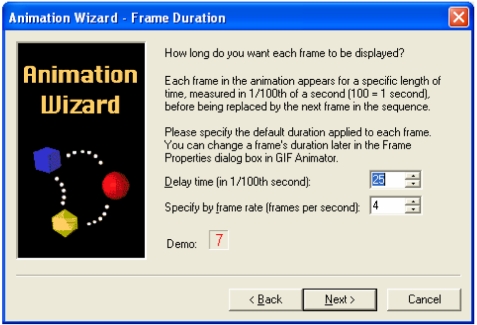
Setting the delay time or the frame rate of the animation.

Step 4 – The user now has to set the duration of each frame to be displayed, by setting either the delay time or the frame rate. A ‘Demo’ field displays an example of the animation speed at the specified delay time or frame rate (Figure 2). The default value of four frames per second is sufficient to produce a smooth animation. Click **Next >** when finished.

**Figure 3:**
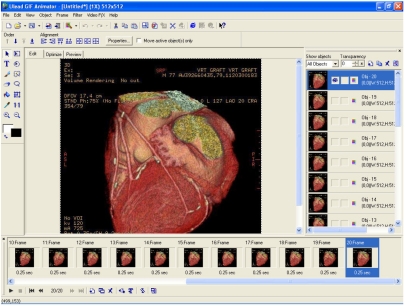
The newly created document following completion of Animation Wizard

Step 5 – Click **Finish** in the final window. The selected images are now compiled in a new document (Figure 3), ready to be saved as an animated GIF or in other formats. The user has the option of saving the output as an animated GIF (.gif), a movie (.avi, .mpg, .mov), or a Macromedia Flash file (.swf).

Prior to exporting the document into desirable formats, it is useful to preview the animation by clicking on the **Preview** tab. The frame rate of an individual image frame may be changed, and a frame may also be dropped or added.

The user may optimise the final output by changing the colour, dither, lossy, and matte values of the image at the **Optimize** tab. This is particularly useful if one is producing an animated GIF as the final file size is dependent on the cumulative size of the images used.

**Figure 4:**
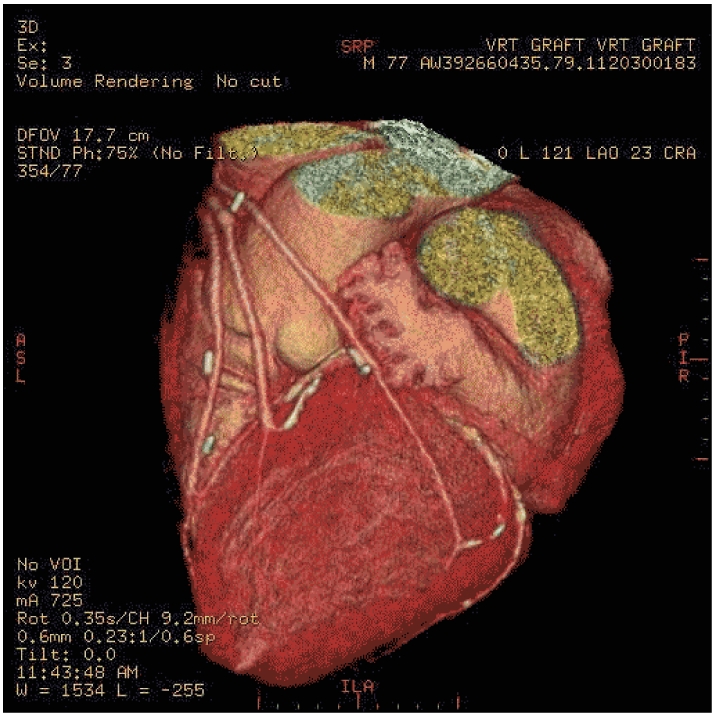
The animated GIF produced by Ulead® GIF Animator^TM^

Figure 4 shows an example of the animated GIF created, which has been optimised using “Preset 32” option under the **Optimize** tab. Note that Ulead® GIF Animator^TM^ accepts both GIF and JPG formats as input images.

The above tutorial is based on Ulead® GIF Animator^TM^ version 5, but may be used with other versions of Ulead® GIF Animator^TM^ with very slight alterations.

## COFFEECUP GIF ANIMATOR

This software is a part of a web design suite of products developed by CoffeeCup Software Inc. (Corpus Christi, TX, USA). For this tutorial, users may either download the software from the above link or download the current version from: http://www.coffeecup.com/. Users may try the software free of cost for 21 days. The current version, CoffeeCup GIF Animator 7.5, is priced at USD 29.00.

Step 1 – Open the software, and click on **File > New.** The Animation Wizard dialog box will appear. Set the length of time for each slide to **0** seconds. Click **Next** when finished.

**Figure 5:**
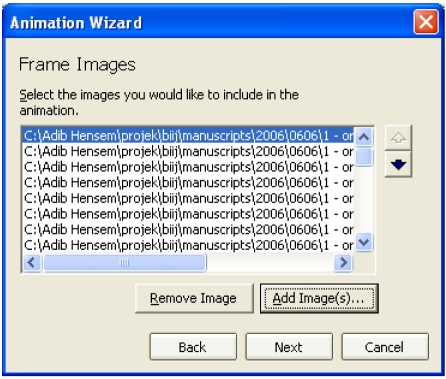
Selecting images to be used in animation

Step 2 – Add individual GIF images by clicking on the **Add Image(s)…** Select the images by first clicking on the file name, followed by **Open**. The selected images will now appear in the previous dialog box (Figure 5). Select multiple images by holding down the Shift key while clicking on the file name (or **Ctrl+A**). Click **Next** when finished.

Step 3 – Click **Finished**, and the user has the option of setting the colour palettes to be used in the **Palettes** window. To select the default settings check the **Make this the Default** option, and click **OK**.

**Figure 6:**
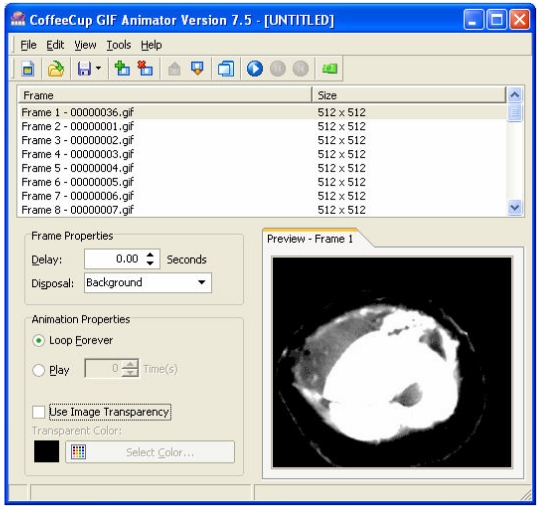
The newly created document containing the selected images

Step 4 – The list of images is now listed in the main software window (Figure 6). Uncheck the “Use Image Transparency” option. The animation may be previewed at the Preview pane by clicking the **Play** **Animation** button (or **Ctrl+P**).

Step 5 – Click **File > Save As…,** and select the location for saving the newly created animated GIF. The user also has the option of saving the output as a Macromedia Flash file (.swf).

**Figure 7:**
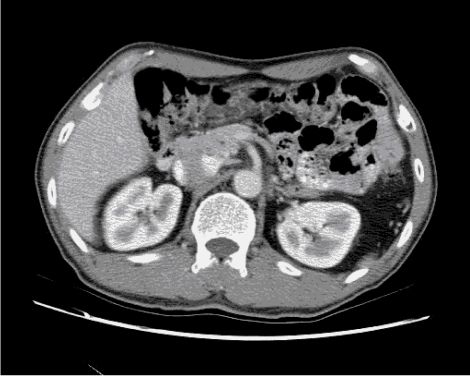
The animated GIF produced by CoffeeCup GIF Animator.

If the user is using the trial version, a small CoffeeCup logo will appear at the top left hand corner in all the animated GIFs created (Figure 7). Note that CoffeeCup GIF Animator only accepts images in GIF format as the input images. The software may also convert AVI movie files into animated GIFs.

The above tutorial is based on CoffeeCup GIF Animator version 7.5, but may be used with other versions of CoffeeCup GIF Animator with very slight alterations.

## MICROSOFT® GIF ANIMATOR

Step 1 – Open Microsoft® GIF Animator, and click on **New** button (or **Ctrl+N**) to create a new document. Click on **Open** button (or Ctrl+O) to add images to the document.

**Figure 8:**
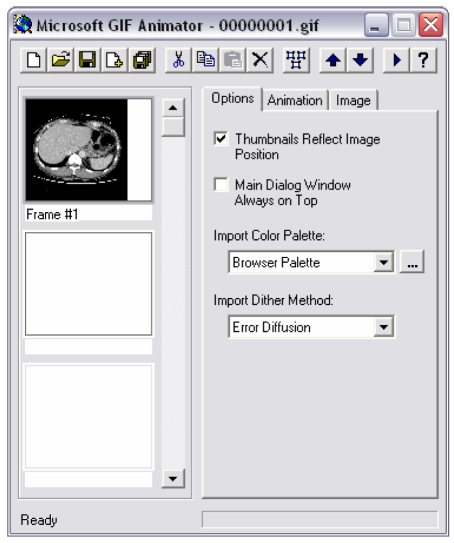
The first image is now inserted into the document.

Step 2 – Select the first image for the animation. Click **Open** when finished. The selected image is now located in the first frame of the animation (Figure 8), on the left side of the window.

Step 3 – Add subsequent images to the document by clicking on **Insert** button (or **Ctrl+I**). Select the subsequent image from the dialog box, and click **Open**.

Step 4 – After all the required images have been selected, the user may preview the output by clicking **Preview** button (or **Ctrl+P**).

**Figure 9:**
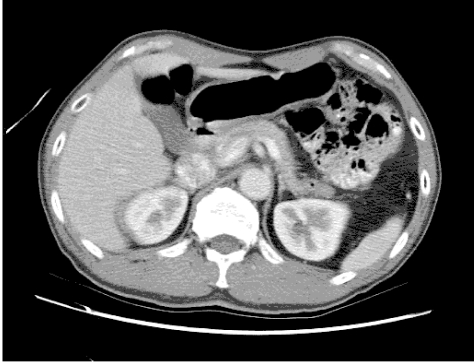
The animated GIF produced by Microsoft® GIF Animator.

Step 5 – Save the file as animated GIF by clicking **Save As…** button (or **Ctrl+A**) and selecting the location. Click **Save** when finished. Figure 9 shows the resulting animated GIF.

To produce a perpetually looping animated GIF, check “Looping” and “Repeat Forever” under the **Animation** tab. Note that Microsoft® GIF Animator only accepts images in GIF format as the input images.
